# Baseline Seroprevalence of Arboviruses in Liberia Using a Multiplex IgG Immunoassay

**DOI:** 10.3390/tropicalmed10040092

**Published:** 2025-04-03

**Authors:** Albert To, Varney M. Kamara, Davidetta M. Tekah, Mohammed A. Jalloh, Salematu B. Kamara, Teri Ann S. Wong, Aquena H. Ball, Ludwig I. Mayerlen, Kyle M. Ishikawa, Hyeong Jun Ahn, Bode Shobayo, Julius Teahton, Brien K. Haun, Wei-Kung Wang, John M. Berestecky, Vivek R. Nerurkar, Peter S. Humphrey, Axel T. Lehrer

**Affiliations:** 1 Department of Tropical Medicine, Medical Microbiology and Pharmacology, John A. Burns School of Medicine, University of Hawai‘i at Mānoa, Honolulu, HI 96813, USA; 2 Department of Biological Sciences, Medical Science, T.J.R Faulkner College of Science and Technology, University of Liberia, Fendall 1000, Liberia; 3 Department of Quantitative Health Sciences, John A. Burns School of Medicine, University of Hawai‘i at Mānoa, Honolulu, HI 96813, USA; 4 National Public Health Institute of Liberia, Monrovia 1000, Liberia; 5 Cell and Molecular Biology Graduate Program, John A. Burns School of Medicine, University of Hawai‘i at Mānoa, Honolulu, HI 96813, USA; 6 Math & Science Department, Kapiolani Community College, University of Hawai‘i at Mānoa, Honolulu, HI 96816, USA

**Keywords:** arbovirus, cross-reactivity, IgG, Liberia, microsphere immunoassay, serosurveillance

## Abstract

Insect-borne viruses may account for a significant proportion of non-malaria and non-bacterial febrile illnesses in Liberia. Although the presence of many arthropod vectors has been documented, the collective burden of arbovirus infections and baseline pre-existing immunity remains enigmatic. Our goal was to determine the seroprevalence of arbovirus exposure across the country using a resource-sparing, multiplex immunoassay to determine IgG responses to immunodominant antigens. 532 human serum samples, from healthy adults, collected from 10 counties across Liberia, were measured for IgG reactivity against antigens of eight common flavi-, alpha-, and orthobunya/nairoviruses suspected to be present in West Africa. Approximately 32.5% of our samples were reactive to alphavirus (CHIKV) E2, ~7% were reactive separately to West Nile (WNV) and Zika virus (ZIKV) NS1, while 4.3 and 3.2% were reactive to Rift Valley Fever virus (RVFV) N and Dengue virus-2 (DENV-2) NS1, respectively. Altogether, 21.6% of our samples were reactive to ≥1 flavivirus NS1s. Of the CHIKV E2 reactive samples, 8.5% were also reactive to at least one flavivirus NS1, and six samples were concurrently reactive to antigens of all three arbovirus groups, suggesting a high burden of multiple arbovirus infections for some participants. These insights suggest the presence of these four arbovirus families in Liberia with low and moderate rates of flavi- and alphavirus infections, respectively, in healthy adults. Further confirmational investigation, such as mosquito surveillance or other serological tests, is warranted and should be conducted before initiating additional flavivirus vaccination campaigns. The findings of these studies can help guide healthcare resource mobilization, vector control, and animal husbandry practices.

## 1. Introduction

The country of Liberia is situated on the lower southwestern tip of West Africa with a population of approximately 5.3 million people living within 15 counties covering 111,370 sq. km. It borders the North Atlantic Ocean to the west, and neighbors Sierra Leone, Guinea, and Côte d’Ivoire [1]. Emerging from more than a decade of civil wars and the recent 2014–15 Ebola virus outbreak, the country is actively engaged in implementing infrastructural improvements to its public health sectors to better manage future biothreats [2]. As part of the pandemic preparedness strategy, active surveillance for human pathogens is being conducted to construct current circulation patterns and natural histories of viral infections in the country. Such comprehensive studies are crucial for guiding government policies, mobilizing healthcare resources, and advising animal husbandry practices in the event of an outbreak.

Given the high number of arbovirus infections in West African nations, mosquito- and tickborne viruses may account for a large proportion of non-malaria and non-bacterial febrile illnesses in Liberia. Such etiologies are severely under-reported as they are frequently undiagnosed, misdiagnosed, or not recognized if asymptomatic [3,4]. The last documented serological study in Liberia, published in 1986, noted a seropositivity rate of 16 and 22% arbovirus-specific antibodies in both epilepsy patients and control participants, respectively [5]. No countrywide survey for common arboviruses has been conducted in Liberia since before the Liberian civil wars. In addition to *Anopheles* sp., many competent *Aedes* and *Culex* sp. vectors are found in Liberia [6]. Considering that the level of urbanization has reached 53% [7] and land use reform has resulted in a deforestation rate of 2% per year prior to 2018 [8], significant landscape changes may contribute to the distribution/adaptation of arbovirus vectors into new habitats, thus expanding the region where the vectors, and the viruses they carry, are found. As mosquito- and tickborne viruses have different lifecycles and reservoir hosts, the ecological niche of these viruses will likely vary across different Liberian counties, from urban and rural communities to coastline regions and neighborhoods bordering other countries.

The purpose of our study is to document the distribution of eight major arboviruses by serology: West Nile virus (WNV), Usutu virus (USUV), Yellow Fever virus (YFV), Dengue virus-2 (DENV-2), Zika virus (ZIKV), Chikungunya virus (CHIKV), Rift Valley Fever virus (RVFV), and Crimean-Congo Hemorrhagic Fever virus (CCHFV). The non-structural protein 1 (NS1) from the flaviviruses, the envelope 2 protein (E2) from CHIKV, the nucleoprotein (N) from RVFV and glycoprotein c (Gc) from CCHFV were selected as analytes for our multiplex immunoassay (MIA) panel due to their immunodominance leading to high assay sensitivity and virus specificity [9,10,11]. Serum samples used in this study represent 10 different counties with varying levels of urbanization, spanning the most populous cities to more rural/forest-covered regions of the country. Additionally, seven collection sites share borders with three other West African countries, which provides valuable information on the regional distribution of these arboviruses.

## 2. Materials and Methods

### 2.1. Ethical Statement and Sample Collection

Protocols for this study were reviewed and approved by the Institutional Review Boards of the University of Hawai‘i at Mānoa, the University of Liberia, as well as the National Research Ethics Review Board of Liberia (NREB). All work was conducted in accordance with institutional and governmental guidelines and regulations. De-identified, archived samples (n = 193) were previously collected from consented, healthy adults (18–80 years of age) in the four counties most heavily affected by the 2014/2015 Ebola Virus Disease outbreak: Montserrado, Margibi, Grand Bassa, and Bong. Additional archived human blood samples (n = 200), from a Lassa Fever study, originating in Bong, Lofa, Nimba, and Grand Bassa county, as well as prospective samples (n = 199) from Grand Cape Mount, Gbarpolu, Grand Gedeh, and River Gee county, were also tested. Approximately 5–10 mL of blood was drawn by venipuncture into BD vacutainer tiger top tubes (BD, Accra, Ghana) from each participant. Sera were separated onsite by centrifugation at 1000–2000× *g* for 10 min and aliquoted into cryovial tubes prior to immediate freezing in a mobile −80 °C freezer. Samples were transported at temperatures below −20 °C to the University of Liberia Fendall Mobile Laboratory for storage at −80 °C until analysis.

### 2.2. Antigen

Stably transformed cell lines expressing the flavivirus (USUV [strain SAAR-1774], DENV-2 [strain TSV01], and ZIKV [strain H/FP/2013]) NS1 and alphavirus (CHIKV [strain Senegal 37997] E2 were generated, generally as previously described [12,13]. Briefly, codon-optimized synthetic genes (IDT, Coralville, IA; and Twist Biosciences, San Francisco, CA, USA) were inserted downstream of an inducible metallothionein (MT) promoter in plasmid vectors pMT-BiP (Invitrogen, Waltham, WA, USA) or pUHM (proprietary). Drosophila S2 cells were transfected with the appropriate selection marker using Lipofectamine-based transfection reagents (Invitrogen, Carlsbad, CA, USA) and cultured in the presence of hygromycin (InvivoGen, San Diego, CA, USA) until stable cell growth was observed. Cell lines were induced with 200 µM copper sulfate, and antigen expression was determined using SDS-PAGE and immunoblot using a 6x-his-tag secondary antibody (Thermofisher Scientific, Waltham, WA, USA). 

Recombinant antigens were purified from clarified cell culture supernatants using a HisTrap FF column (Cytiva, Marlborough, MA, USA). Optimized purification protocols, using a single or double imidazole elution step, were developed for each antigen to achieve maximum purity. Select fractions containing the antigen were pooled, buffer-exchanged into PBS, and stored at −80 °C until use. Purity for all internally produced antigens was ≥90% as estimated by SDS-PAGE.

Drosophila S2-expressed WNV NS1 and CCHFV Gc were gifted by Hawai‘i Biotech Inc. (Honolulu, HI, USA), and YFV NS1 (cat no. YFV-NS1-100) and RVFV N (cat no. REC31640-100) were purchased from the Native Antigen Company (Oxford, UK). The reactivity of each antigen was confirmed using a panel of arbovirus IgG reference sera (provided by the Vector Borne Disease Branch of the US Centers for Disease Control and Prevention, and the United States Department of Agriculture) by Western blot.

### 2.3. Arbovirus Microsphere Panel

Antigens were coupled to spectrally distinct MagPlex-C Microspheres (Luminex Corporation, Austin, TX, USA) using a modified, two-step carbodiimide reaction based on the manufacturer’s protocol. A total of 25 µg of each antigen was covalently coupled to the surface of 5 × 10^6^ microspheres. The antigen-conjugated microspheres were stored in 800 µL of PBS-TBN buffer (PBS with 0.1% bovine serum albumin Fraction V, 0.02% Tween-20, and 0.01% sodium azide (Sigma-Aldrich, St. Louis, MO, USA) at 2–8 °C until use.

### 2.4. Microsphere Immunoassay

IgG seroreactivity of human serum samples to each of the eight arbovirus antigens and BSA as the negative antigen control was measured using a multiplex microsphere-based immunoassay (MIA) as described previously [13]. The MagPix instrument was calibrated with reference beads prior to each use. Serum samples were initially diluted to 1:100 with PBS+ 1% BSA and 0.02% Tween 20 (PBS-BT) and added to the arbovirus microsphere panel (1:200 suspended in PBS-BT) 1:1 in black-sided 96-well plates in duplicates. The antibody-bead complex was allowed to form for 3 h at ambient temperature (32–35 °C) with agitation. After two PBS-BT wash steps, 50 μL of 1 μg/mL red phycoerythrin (R-PE)-conjugated goat anti-human IgG antibodies (Jackson ImmunoResearch, Inc., West Grove, PA, USA) were added to each well and incubated at ambient temperature with agitation for 1 h. Finally, after another series of wash steps and resuspension in MAGPIX^®^ drive fluid, the median fluorescence intensity (MFI) was measured using a MAGPIX^®^ Instrument (Luminex Corporation, Austin, TX, USA). Samples with coefficient of variation percent (CV%) values of >15% or with low bead counts (below 50 beads per region) were retested.

### 2.5. Statistical Analysis

Samples with high background, i.e., a MFI of 1500 or greater for BSA and all other analytes, were removed from further analysis. MFI cutoff for high seroreactivity was determined using a Gaussian mixture model to identify clustering of low, medium, and high MFIs [14]. Values were log-transformed, and the maximum likelihood estimation for normal clusters of a specified number of components was determined using the Expectation-Maximization (EM) algorithm within the (mixR) package on R software (v 4.3.1) [15]. The number of components for each distribution was increased until a clear high MFI cluster was distinguishable. The vertical intercept between the left tail of the high MFI and the right tail of the adjacent normal curves was set as the cutoff for highly seroreactive samples (Figure S1A). The assay background was determined using the mean MFI of BSA + 3 standard deviations. Individual antigen analyte cutoffs of low-medium seroreactivity were determined using the mean MFI of samples showing no specific reactivity to any analyte + 3 standard deviations. Although not available for every analyte, the cutoff demarcating highly seroreactive samples was verified using positive reference sera.

To resolve the signal interference caused by cross-reactivity between the USUV, WNV, YFV, DENV-2, and ZIKV NS1s, ratios for each analyte were generated by dividing each of the flavivirus NS1 MFIs by the NS1 MFIs of interest for each participant sample [9]. This yielded a single relative MFI (rMFI) ratio of 1.0, where the numerator and denominator values are the same analyte, and four other ratios of either <1.0 or >1.0 for the other flavivirus NS1 analytes (Figure S1B). Samples with monotypic reactivity for a single flavivirus NS1 had a ratio of 1.0 for the analyte corresponding to the probable etiology of the primary infection and had relative ratios of <0.5 for the other four flavivirus NS1 analytes. Samples with reactivity to two or more flavivirus NS1s, defined as having two rMFI ratios ≥ 1.0, were categorized as having had multiple flavivirus infections. To determine the distribution of flavivirus infections by county (Table 1), only the strongest flavivirus NS1 response was considered. In the case of samples having multiple NS1 reactivities above the established cutoff, the NS1 analyte with the highest MFI was selected, while the other NS1 MFI values were removed from the tally. Only a single NS1 analyte contributed to the final count. Samples with multiple flavivirus NS1 rMFI ratios >1 were enumerated and counted separately (Table 2).

Graphical representation was plotted using Prism, GraphPad Software (v10.0.1), (San Diego, CA, USA).

## 3. Results

**Figure 1:**
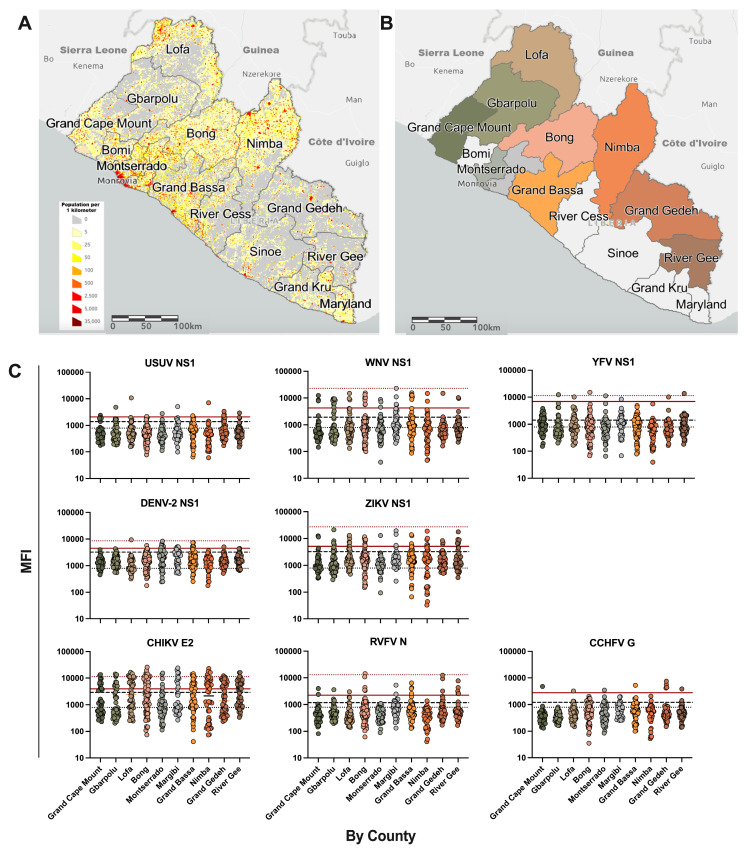
Distribution of arbovirus seroreactivity in 10 counties across Liberia. (**A**) Population distribution of Liberia per square kilometer. The image was generated with ArcGIS Online, using the 2016 Liberian Population Density layer, generated by the USAID Liberia, available on the online service. (**B**) Selected counties where participant serum samples originated. Each selected county is displayed in a different color. (**C**) Curated distribution of arbovirus antigen reactivities by county. The black dotted line indicates the background signal of BSA + 3 STDEV. The black dashed line indicates the analyte-specific assay cutoff determined by using the mean MFI of samples showing no specific reactivity to any analyte + 3 STDEV. The red solid line indicates the Gaussian mixture model cutoff for highly seroreactive samples. Where available, the red dotted line indicates the MFI value of a PCR-confirmed, arbovirus-positive reference sera.

Archived samples from 393 healthy adult participants from Montserrado, Margibi, Grand Bassa, Bong, Nimba, and Lofa counties were collected in 2021 to survey the seroprevalence of Ebola and Lassa virus after the 2014 Ebola Virus Disease and annual Lassa Fever outbreaks. To gain greater resolution and to provide a contrast to the urban counties with large population centers (Figure 1A), 199 samples were prospectively collected from participants inhabiting Grand Cape Mount, Gbarpolu, Grand Gedeh, and River Gee counties. These four counties are comprised primarily of rural communities, with lower population densities, and border the countries of Sierra Leone, Guinea, and Côte d’Ivoire (Figure 1A,B).

**Table 1:** Individual Count, Percentage by County and Countrywide Total of Highly Seroreactive Samples to Each Arbovirus Antigen.

County	n	USUV NS1	WNV NS1	YFV NS1	DENV-2 NS1	ZIKV NS1	RVFV N	CHIKV E2	CCHFV Gc
Grand Cape Mount	49	1 (2.0%)	3 (6.1%)	-	-	2 (4.1%)	2 (4.1%)	13 (26.5%)	1 (2.0%)
Gbarpolu	50	1 (2.0%)	9 (18.0%)	1 (2.0%)	-	4 (8.0%)	1 (2.0%)	12 (24.0%)	-
Lofa	50	1 (2.0%)	3 (6.0%)	1 (2.0%)	1 (2.0%)	5 (10.0%)	1 (2.0%)	22 (44.0%)	1 (2.0%)
Bong	79	1 (1.3%)	8 (10.1%)	1 (1.3%)	1 (2.0%)	5 (6.3%)	5 (6.3%)	29 (36.7%)	-
Montserrado	51	1 (2.0%)	3 (5.9%)	1 (2.0%)	7 (13.7%)	1 (2.0%)	-	5 (9.0%)	1 (2.0%)
Margibi	40	1 (2.5%)	4 (10.0%)	1 (2.5%)	2 (5.0%)	3 (7.5%)	2 (5.0%)	11 (27.5%)	-
Grand Bassa	66	1 (1.5%)	6 (9.1%)	-	4 (6.1%)	3 (4.5%)	4 (6.1%)	20 (30.3%)	1 (1.5%)
Nimba	48	1 (2.1%)	2 (4.2%)	-	-	6 (12.5%)	-	21 (43.8%)	-
Grand Gedeh	49	3 (6.1%)	1 (2.0%)	1 (2.0%)	1 (2.0%)	4 (8.2%)	3 (6.1%)	17 (34.7.0%)	4 (8.2%)
River Gee	50	1 (2.0%)	2 (4.0%)	1 (2.0%)	1 (2.0%)	5 (10.0%)	5 (10.0%)	23 (46.0%)	1 (2.0%)
Countrywide Total	532	12	41	7	17	38	23	173	9
Percentage	-	2.3%	7.7%	1.3%	3.2%	7.1%	4.3%	32.5%	1.7%

- indicates no seroreactivity detected.

**Table 2:** Probable count, percentage by county, and countrywide total of single and multiple arbovirus infections.

County	n	Single Flavivirus Infection	Multiple (≥2) Flavivirus Infections	Total Flavivirus Infection	Single Flavivirus w/CHIKV Infection	Multiple Flavivirus w/CHIKV Infection	CHIKV with RVFV Infection	CHIKV with CCHFV Infection
Grand Cape Mount	49	6 (12.2%)	-	6 (12.2%)	2 (4.1%)	-	-	-
Gbarpolu	50	13 (26.0%)	2 (4.0%)	15 (30.0%)	6 (12.0%)	-	1 (2.0%)	-
Lofa	50	9 (18.0%)	2 (4.0%)	11 (22.0%)	4 (8.0%)	-	1^s^ (2.0%)	-
Bong	79	11 (13.9%)	5 (6.3%)	16 (20.3%)	8 (10.1%)	4 (5.1%)	3 (1^s^) (3.8%)	-
Montserrado	51	12 (23.5%)	1 (2.0%)	13 (25.5%)	1 (2.0%)	1 (2.0%)	-	1^m^ (2.0%)
Margibi	40	7 (17.5%)	4 (10.0%)	11 (27.5%)	-	2 (5.0%)	1 (2.5%)	-
Grand Bassa	66	11 (16.7%)	3 (4.5%)	14 (21.2%)	4 (6.1%)	2 (3.0%)	-	1^m^ (1.5%)
Nimba	48	9 (18.8%)	-	9 (18.8%)	4 (8.3%)	-	-	-
Grand Gedeh	49	7 (14.3%)	3 (6.1%)	10 (20.4%)	2 (4.1%)	2 (4.1%)	-	3 (6.1%)
River Gee	50	10 (20.0%)	-	10 (20.0%)	3 (6.0%)	-	2 (1^s^) (4.0%)	1^s^ (2.0%)
Countrywide Total	532	95	20	115	34	11	8	6
Percentage	-	17.9%	3.8%	21.6%	6.4%	2.1%	1.5%	1.1%

- indicates no seroreactivity detected. n^s^ indicates the number of people with a single flavivirus infection and n^m^ indicates the number of people with multiple flavivirus infections with CHIKV and either RVFV or CCHFV infections.

Of the 592 serum samples tested, 60 samples were removed from the analysis due to excessively high background signals and non-specific binding to all analytes, including BSA, which is used as a negative analyte control and is present in all assay buffers. Samples removed from the analysis showed either signs of hemolysis or had high lipid content; however, a few samples were aberrantly reactive without any visible signs of compromised sample integrity. A total of 532 samples yielded consistent MFI values with a low background signal. Gaussian mixture modeling of log-transformed MFI values was used to identify low, medium, and high MFI clusters that were used to establish cutoffs distinguishing highly seroreactive samples apart from adjacent lower MFI clusters (Figure S1A). Samples with MFIs greater than the cutoff value were considered highly seroreactive and likely to have antigen-specific antibodies for the detected analyte.

Cross-reactivity between the flavivirus NS1s (homology ranging from 45 to 76%), which yields MFIs above the set cutoffs for multiple flavivirus analytes, could result in duplicate counting. This was controlled for by using rMFI ratios, where the MFI of each flavivirus NS1 analyte is divided by the MFI of the NS1 of interest (Figure S1B). Reactivity to a single NS1 analyte indicates that a participant has had only a primary flavivirus infection, whereas reactivity to two or more NS1 analytes suggests multiple flavivirus infections. Table 1 displays the total count of sample reactivities to all arbovirus analytes countrywide and sorted by county. Here, only the tally of single flavivirus was counted. In the event a serum sample is reactive to two or more flavivirus NS1s, the analyte with the highest ratio was selected for the final count, and its corresponding MFI readouts for the other flavivirus NS1s were removed from the tally.

Overall, a third of our serum samples (32.5%) were reactive to the CHIKV E2, suggesting a moderate seroprevalence for alphaviruses in the Liberian population. This was followed by reactivities to WNV NS1 at 7.7% and ZIKV NS1 at 7.1%. Lower seroprevalence was observed for RVFV N at 4.3% and DENV-2 NS1 at 3.2%. While at much lower rates, IgG against USUV NS1, YFV NS1, and CCHFV Gc were also detected. The presence of these antigen-specific antibodies suggests that at least eight arboviruses may have or are currently circulating in Liberia.

Table 2 displays the count of samples with single flavivirus and multiple arbovirus reactivity. Approximately 17.9% of our samples were reactive to only a single flavivirus analyte, while 3.8% were reactive to two or more flavivirus NS1s. Co-reactivities for both DENV-2 and ZIKV NS1s were the most common. Altogether, over a fifth of our samples (21.6%) were reactive to at least one flavivirus NS1. Of the samples reactive to CHIKV E2, 6.4% and 2.1% were also reactive to either a single or multiple flavivirus NS1(s), respectively, for a total of 8.5% co-exposure. Similarly, approximately 1–2% of CHIKV E2 reactive samples were also reactive to either RVFV N or CCHFV Gc, and six samples were reactive to at least one analyte from three virus families with no pattern based on location.

## 4. Discussion

This report provides the first published update on the epidemiological situation of arbovirus infections in Liberia since 1986 [5]. Our pilot study uses a multiplex immunoassay based on serum reactivity to immunodominant, recombinant antigen from eight highly relevant arboviruses, and a statistical modeling approach using MFI signal clustering, to determine if the spatial differences in arbovirus antigen reactivity can be seen in 532 archived and prospective serum samples collected from 10 counties.

Overall, arbovirus infections appear to be widespread throughout the country. Approximately 60% of our samples were reactive to at least one arbovirus analyte. Notably, there is a moderate to low degree of alphavirus and flavivirus seroprevalence. The most prevalent infection appears to be from an alphavirus (CHIKV), followed by WNV, ZIKV, RVFV and finally DENV-2; altogether suggesting endemic circulation of these arboviruses in Liberia. There is also a low rate of dual reactivity to antigens from both alphaviruses and flaviviruses in some samples; with six samples reactive to antigens of three arbovirus families, indicating a potentially heavy burden of mosquito-borne diseases for a few individuals. After accounting for possible cross-reactivity, evidence of YFV, USUV, and CCHFV infections was found, with approximately one sample per county, with some exceptions, seroreactive to these antigens; however, it is not clear whether this indicates active endemic transmission rather than cross-border transmission from neighboring countries [16,17,18]. The primary vectors for all these arboviruses (*Aedes aegypti*, *Culex pipiens*, *Hyalomma* spp., respectively) are present in Liberia [6,19].

The arboviral landscape of Liberia reported here aligns with large-scale, mosquito-borne disease outbreaks and serological evidence of flavi-, alpha-, and orthobunya-/nairoviruses documented in this region [20,21,22,23,24,25,26,27,28,29,30,31]. Antibodies to CHIKV have been reported in individuals from Liberia and Sierra Leone since 1977 [32], and was cited as a cause of acute febrile illnesses in Sierra Leone [30] and Guinea [33]. Our report of 32.5% seroprevalence for CHIKV is consistent with the 31.2% reported in Mali [34], and the 38.5–54% reported in Senegal [35,36]. CHIKV E2 reactivity may also be attributed to, or at least in part, by O’nyong’nyong virus (ONNV), a closely related but divergent alphavirus, transmitted by *Anopheles* mosquitoes [37]. The E2 antigen of CHIKV and ONNV shares 87% homology. Neutralization tests performed with ONNV on CHIKV IgM+ samples in Sierra Leone suggest that ONNV may be predominant in West Africa [30], and a 2003 outbreak in neighboring Cȏte d’Ivoire makes this notion highly plausible that ONNV cross-reactivity may contribute to the high rate of CHIKV E2 reactivity in our assay [38]. While we attempted to include ONNV E2 in our analysis, the purity of our antigen was ~25%, which resulted in a high background signal in our assay. Together with the high homology, we could not establish a clear antigen cutoff and thus excluded the ONNV data set from our final analysis.

By contrast, the IgG seroprevalence to WNV (7.7%) and ZIKV (7.1%) NS1s in our samples are lower than the rates reported in other countries; however, many of the seroprevalence rates reported in the literature are derived from febrile or clinic-based patient samples and cannot be directly compared. The prevalence rates of WNV antibodies in febrile patients in Mali were reported to be 39% [39], and upwards of 40% in Sierra Leone [40]. In healthy children and adults in Ghana, the rate of WNV-specific IgG was ~30% overall. By comparison, ZIKV seroprevalence in Mali was 12% [41] and 25.8% [34] for afebrile and febrile participants, respectively, while the average IgM seroprevalence of febrile patients in Guinea was about 14.7% [42].

While RVFV has been detected in animals in Senegal, Guinea, and Cȏte d’Ivoire [43,44,45,46], the prevalence of infection in humans appears to vary depending on the region sampled [47] and on the occupation of the individual, with higher infection rates found in those working with livestock [48]. The overall seroprevalence of RVFV in Africa was reported as 7.9% [49] and is comparable to our rate of 4.3%.

Molecular evidence of all four DENV serotypes has been recorded in Senegal, Burkina Faso, Ghana, Sierra Leone, and Cȏte d’Ivoire, where the viruses are endemic [20,31,50,51,52,53,54,55]. Similarly, much of the seroprevalence of DENV reported in the literature is derived from febrile patients; however, the distribution of these infections is likely heterogeneous depending on the region sampled [56]. Between 69.2 and 78.6% of febrile hospital patients in Ghana and Sierra Leone were determined to have been infected with DENV [57,58]. One study in Mali, via random household sampling, reported a seroprevalence rate of 77.2% based on virus neutralization tests [34]. Comparatively, the DENV-2 NS1 seroprevalence rate of 3.2% in our study is much lower. The higher rates for flavivirus NS1 reactivity in the literature could be partially attributed to the difficulty in distinguishing flavivirus cross-reactivity, especially in the case of multiple flavivirus infections, using commercial ELISA kits. Furthermore, our DENV NS1 analyte, derived from DENV-2, may not capture the full extent of all DENV serotype infections, especially from low-titer convalescent samples.

It is evident, though, that highly DENV-2 NS1 reactive samples appeared to largely cluster in the central coastal counties of Liberia with the majority coming from Montserrado and Grand Bassa counties, which have urban or semi-urban settings along the Atlantic Ocean. The lower coastal elevation, slightly warmer climate, and contribution of salinity-adapted *Aedes aegypti* may explain the urban distribution of DENV-2 observed in this study [59,60]. This mosquito species is the primary vector for DENV and is found commonly in anthropophilic settings [6]. It is also noted that no RVFV N reactive samples were detected from Montserrado County, which supports the observation that RVFV is primarily a rural disease [61]. While both *Aedes* and *Culex* spp. are susceptible to RVFV infection, transmission by *Aedes aegypti* appears to be host-dependent [61,62] and unlikely in an urban setting, as ruminant hosts are less likely to be present [61]. Similarly, CHIKV reactive samples were more prevalent outside of Montserrado County and may be attributed to the greater presence of *Aedes albopictus,* which is typically found in rural settings [63], and is a more competent vector for CHIKV than *Aedes aegypti* [64]. The dichotomy of DENV-2 and CHIKV seroprevalence may be explained by the preferred habitats of these two *Aedes* spp. [65]. Although less prominent, a similar trend was observed with ZIKV infections, as only a single reactive sample originated in Montserrado County. The contribution of CHIKV and ZIKV transmission from the sylvatic cycle by *Aedes albopictus* and *Aedes africanus* is a possible factor for their predominance in rural settings [25,66]. In terms of total flavivirus infections, there appears to be an even spread across the country, whereas individuals with both flavivirus and alphavirus (CHIKV) infection appear more frequently outside the urbanized Montserrado and Margibi counties. Lastly, there is a trend of more WNV infections occurring west of Nimba County. Although *Aedes* spp. are competent vectors for WNV transmission, *Culex* spp., especially *Culex quinquefasciatus*, predominate as the primary source of transmission in Africa [67]. This seroprevalence pattern suggests that the distribution of arboviruses may be determined by the type of mosquito hosts that inhabit certain ecological niches created by terrain, climate, and level of urbanization.

Precautions were taken to ensure valid measurements and accurate classification within our data set, however, there are some limitations to be considered. (1) A portion of samples, especially those archived, were deidentified or lost, and thus, sociodemographic data could not be analyzed to establish potential risk factors. (2) Systematic household sampling was not possible in every circumstance; thus, a portion of sera were collected on a voluntary basis at a single community center in the county. This sampling bias could subtly influence our results, as some samples were collected only from those who could travel to the collection site. However, our non-healthcare-based sample pool of healthy adults may capture asymptomatic and underreported cases that would otherwise be missed. (3) Although most of our samples yielded clear readouts with low background interference, we had to exclude some samples during our quality control screening due to poor sample integrity. This reduced our sample size by 10%. Timely sample processing is critical for settings where access to laboratory equipment is not feasible within a few hours after collection. It is recommended that blood samples be processed as soon as possible and immediately transported/stored between −20 and to −80 °C. (4) Our assay relied on IgG reactivity to immunodominant antigens. While this enables better specificity compared to traditional ELISA methods and allows for evaluation of antibody binding in the context of other similar antigens, NS1 cross-reactivity still limits the resolution required to distinguish the etiology of multiple flavivirus infections. Virus neutralization tests will be required to corroborate our results; however, this is exceedingly difficult to accomplish in-country due to the lack of appropriately equipped biocontainment facilities, assay reagents, and skilled personnel. The utility of this microsphere immunoassay stems from its high-throughput capacity to quickly and widely survey for the presence of antibodies in humans or animals [68] against certain pathogens as routine disease surveillance or in response to an outbreak situation. As IgG binding-based assays are not as definitive as neutralization tests, the addition of *Aedes* and *Culex* spp. to ongoing mosquito surveillance can help corroborate the presence of these specific arboviruses in the country. (5) Lastly, our YFV NS1 analyte is unable to discriminate wt-YFV infection from responses generated with vaccination-attenuated YFV-17D. While the countrywide YF vaccination coverage had dropped below 60% between 2014 and 2015, current coverage is estimated to be ~78–92% in 2022. Our results can only be interpreted as baseline YFV immunity rather than the prevalence of YFV infections.

## 5. Conclusions

Our analysis provides a critical update of baseline arbovirus immunity in Liberia. We show low and moderate rates of flavi- and alphavirus infections, respectively, in healthy adults, with more DENV-2 seroprevalence in urban counties and ZIKV and CHIKV seroprevalence in rural counties. The seroreactivity rates for RVFV and CCHFV are also low relative to other African countries. This alludes to the role of various mosquito vectors in arbovirus distribution, thus careful speciation of *Aedes*, *Culex*, and *Anopheles* (for ONNV) mosquitos may provide further insight into virus transmission patterns. Arbovirus-specific confirmational tests and/or mosquito surveillance should be conducted before the initiation of mass vaccination campaigns in the country. The findings of these studies can further help guide healthcare resource mobilization, vector control, and animal husbandry practices.

## Data Availability

Date will be provided upon reasonable request by the corresponding authors, Albert To (albertto@hawaii.edu), and Axel T. Lehrer (lehrer@hawaii.edu).

## Supplementary Materials

The following supporting information can be downloaded at: https://www.mdpi.com/article/10.3390/tropicalmed10040092/s1, Figure S1: Distribution of Arboviruses in 10 Counties across Liberia.
