# Supplementary material for: Baseline Seroprevalence of Arboviruses in Liberia Using a Multiplex IgG Immunoassay
Source: Trop Med Infect Dis. 2025 Apr 3;10(4):92. doi: 10.3390/tropicalmed10040092 (PMC12031126; doi:10.3390/tropicalmed10040092)
Supplement: Supplementary file 1 [file tropicalmed-10-00092-s001.zip › tropicalmed-3465979-supplementary.pdf]

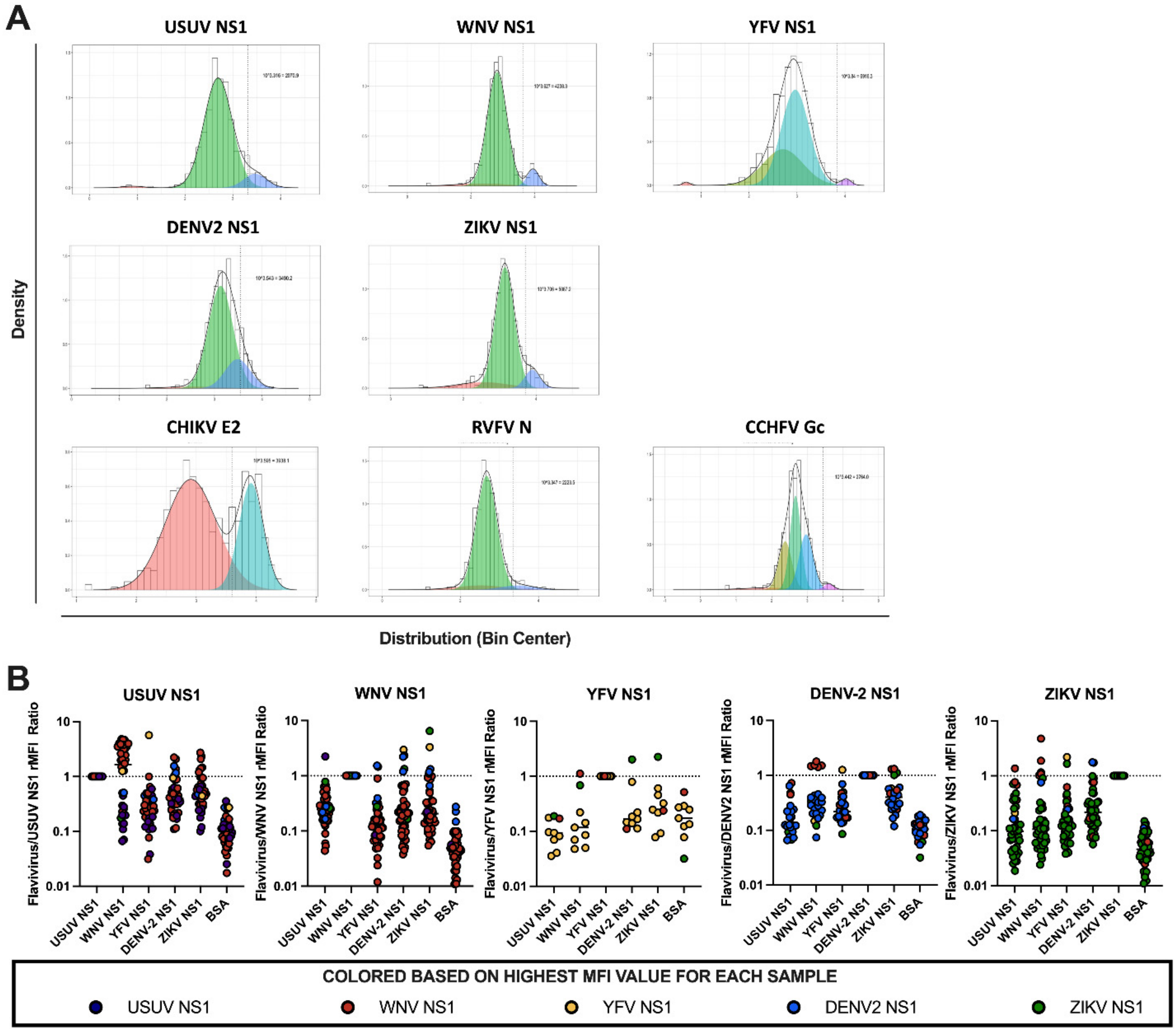

Figure S1. Distribution of arboviruses seroreactivity in 10 counties across Liberia. (**A**) Clustering of low, medium and high MFIs per analyte were grouped using a Gaussian Mixture Model on R software. Normal clusters of a specified number of components were determined using the Expectation-Maximization (EM) algorithm. The vertical intercept between the left tail of the high MFI and the right tail of the adjacent normal curves was used to determine the cutoff for highly seroreactive samples. Samples with BSA MFIs of over 1500 were excluded from the analysis. (**B**) Flavivirus NS1 relative MFI (rMFI) Cross-Reactivity Ratios. Ratios were generated by dividing the MFI from each flavivirus NS1 analyte by the NS1 MFI of interest (e.g. WNV/WNV NS1. WNV/USUV NS1, etc.) Each sample was assigned a color corresponding to the analyte with the highest MFI. Ratios < 1.0 are typically below 0.5 for samples from individuals who have experienced a primary infection with the denominator. A ratio > 1.0 indicates that the observed MFI, may be the result of cross-reactivity. In this case only the data point for the analyte with the highest MFI is kept and the others are removed from the final tally.
